# Poly(A) Binding Protein Is Required for Nuclear Localization of the Ecdysteroidogenic Transcription Factor Molting Defective in the Prothoracic Gland of *Drosophila melanogaster*

**DOI:** 10.3389/fgene.2020.00636

**Published:** 2020-06-26

**Authors:** Takumi Kamiyama, Wei Sun, Naoki Tani, Akira Nakamura, Ryusuke Niwa

**Affiliations:** ^1^ Graduate School of Life and Environmental Sciences, University of Tsukuba, Tsukuba, Japan; ^2^ Laboratory of Evolutionary and Functional Genomics, School of Life Sciences, Chongqing University, Chongqing, China; ^3^ Life Science Center for Survival Dynamics, Tsukuba Advanced Research Alliance (TARA), University of Tsukuba, Tsukuba, Japan; ^4^ Department of Germline Development, Institute of Molecular Embryology and Genetics, Kumamoto University, Kumamoto, Japan

**Keywords:** poly(A) binding protein, nuclear localization, insect development, ecdysone biosynthesis, Halloween gene

## Abstract

Steroid hormone signaling contributes to the development of multicellular organisms. In insects, ecdysteroids, like ecdysone and the more biologically-active derivative 20-hydroxyecdysone (20E), promote molting and metamorphosis. Ecdysone is biosynthesized in the prothoracic gland (PG), *via* several steps catalyzed by ecdysteroidogenic enzymes that are encoded by Halloween genes. The spatio-temporal expression pattern of ecdysteroidogenic genes is strictly controlled, resulting in a proper fluctuation of the 20E titer during insect development. However, their transcriptional regulatory mechanism is still elusive. A previous study has found that the polyadenylated tail [poly(A)] deadenylation complex, called Carbon catabolite repressor 4-Negative on TATA (CCR4-NOT) regulates the expression of *spookier* (*spok*), which encodes one of the ecdysteroidogenic enzymes in the fruit fly *Drosophila melanogaster*. Based on this finding, we speculated whether any other poly(A)-related protein also regulates *spok* expression. In this study, we reported that poly(A) binding protein (Pabp) is involved in *spok* expression by regulating nuclear localization of the transcription factor molting defective (Mld). When *pabp* was knocked down specifically in the PG by transgenic RNAi, both *spok* mRNA and Spok protein levels were significantly reduced. In addition, the *spok* promoter-driven green fluorescence protein (GFP) signal was also reduced in the *pabp*-RNAi PG, suggesting that Pabp is involved in the transcriptional regulation of *spok*. We next examined which transcription factors are responsible for Pabp-dependent transcriptional regulation. Among the transcription factors acting in the PG, we primarily focused on the zinc-finger transcription factor Mld, as Mld is essential for *spok* transcription. Mld was localized in the nucleus of the control PG cells, while Mld abnormally accumulated in the cytoplasm of *pabp*-RNAi PG cells. In contrast, *pabp*-RNAi did not affect the nuclear localization of other transcription factors, including ventral vein lacking (Vvl) and POU domain motif 3 (Pdm3), in PG cells. From these results, we propose that Pabp regulates subcellular localization in the PG, specifically of the transcription factor Mld, in the context of ecdysone biosynthesis.

## Introduction

Ecdysteroids, like ecdysone and the more biologically-active derivative 20-hydroxyecdysone (20E), regulate several biological events in insects (Niwa and Niwa, 2014; Uryu et al., 2015). The 20-hydroxyecdysone titers are temporally changed during insect development, where proper fluctuations of 20E titers are essential for insect development (Riddiford, 1993; Nijhout, 1994; Bellés, 2020). Ecdyone is biosynthesized in an endocrine organ named the prothoracic gland (PG) *via* several steps catalyzed by step-specific enzymes (Niwa and Niwa, 2014). The enzymes are encoded by a group of genes often called the Halloween gene (Rewitz et al., 2007). Therefore, the regulation of ecdysteroidogenic genes expression is essential to achieve proper fluctuations of 20E titers (Niwa and Niwa, 2016). However, the molecular mechanism by which the expression of ecdysteroidogenic genes is regulated is yet to be fully elucidated.

Previously, we have reported that polyadenylated tail [poly(A)] degradation complex, called Carbon catabolite repressor 4-Negative on TATA (CCR4-NOT) is involved in the regulation of ecdysteroidogenic gene expression in the fruit fly *Drosophila melanogaster* (Zeng et al., 2018). By knocking down the gene *phosphoglycerate kinase promoter directed over production* (*Pop2*), which encodes a vital component of the CCR4-NOT complex, specifically in the PG, *D. melanogaster* animals show a larval-arrested phenotype. Furthermore, the expression levels of some ecdysteroidogenic genes are strongly decreased in these animals. Based on this finding, we hypothesized that other poly(A) related protein(s) may also contribute to the expression of ecdysteroidogenic genes.

In this study, we revealed that poly(A) binding protein (Pabp) contributes to the expression of ecdysteroidogenic genes. The knockdown of the *pabp* gene in the PG caused first instar-arrest and a decrease in ecdysteroidogenic gene expression, especially *spookier* (*spok*). Interestingly, the nuclear localization of the transcription factor molting defective (Mld), a transcriptional activator of *spok*, was disrupted in PG cells of *pabp*-RNAi larvae. Our results suggest that Pabp positively regulates ecdysteroidogenic gene expression by regulating ecdysteroidogenic transcription factors.

## Materials and Methods

### *D. melanogaster* Strains

*D. melanogaster* flies were reared on a standard agar-cornmeal medium at 25 or 17°C under a 12:12 h light/dark cycle. *w* ^1118^ served as a control strain. *phm-GAL4#22* (a gift from Michael B. O’Connor, University of Minnesota, MN; McBrayer et al., 2007; Yamanaka et al., 2013) was used as the strain to drive forced gene expression in the PG. *UAS-dicer2* (#24650) was obtained from the Bloomington *Drosophila* Stock Center. *UAS-pabp-IR* (#22007) and *UAS-mld-IR* (#17329) were obtained from the Vienna *Drosophila* Resource Center. Transgenic RNAi experiments were conducted by crossing these *UAS RNAi* lines with *w*, *UAS-dicer2*, and *phm-GAL4#22/TM6 Ubi-GFP*. A strain carrying the 1.45 kb *spok* promoter-fused GFP cassette (*spok>GFP*) was previously described (Komura-Kawa et al., 2015).

### Generating Anti-Mld Antibody

Rabbit polyclonal anti-Mld antibody was raised against the peptide: NH_2_-MSANRRRRSASAASSIAAET-COOH, which corresponds to 1–20 amino acid residues of the mature Mld protein (Neubueser et al., 2005).

### Immunostaining

Immunostaining of prothoracic glands was performed as described previously (Imura et al., 2017). Briefly, dissected larval tissues were fixed in 3.7% formaldehyde in phosphate-buffered saline (PBS) for 30 min at room temperature. Samples were then washed with PBS + 0.3% Triton X-100 (Nacalai tesque, Kyoto Japan) and incubated overnight at 4°C with primary antibodies: rabbit anti-Mld antibody (1:200), guinea pig anti-*Spok* antibody (1:200; Gibbens et al., 2011), rabbit anti-Phantom (Phm) antibody (1:200; Parvy et al., 2005), rat anti-Ventral vein lacking (Vvl) antibody (1:3,000; Anderson et al., 1995), and guinea pig anti-POU domain motif 3 (Pdm3; 1:100; Chen et al., 2012). As fluorescent secondary antibodies, we used goat anti-guinea pig Alexa Fluor 488 (Life Technologies, Carlsbad, CA, USA) and goat anti-rabbit Alexa Fluor 555 (Life Technologies, Carlsbad, CA, USA). The secondary antibodies were diluted 1:200 and incubated for 2.5 h at room temperature. Nuclear stains used in this study were 4',6-diamidino-2-phenylindole (DAPI; Sigma-Aldrich, St. Louis, MO, USA) and TOPRO3 (Thermo Fisher Scientific, Waltham, MA, USA). For DAPI staining, after the incubation with the secondary antibodies, the samples were washed and then incubated with 1 μg/ml (final concentration) of DAPI for 1 h. For TOPRO3 staining, the samples were incubated with the secondary antibodies along with 10 μg/ml (final concentration) of RNase A (Takara Bio, Kusatsu, Japan). The samples were then washed, followed by the 1-h incubation with 10 μm TOPRO3 (Thermo Fisher Scientific, Waltham, MA, USA). Confocal images were captured using the LSM 700 laser scanning confocal microscope (Carl Zeiss, Oberkochen, Germany). Images were processed using the ImageJ software (Schneider et al., 2012).

### Quantitative Reverse Transcription-Polymerase Chain Reaction

RNA was isolated from the whole bodies of the second instar larvae using the RNAiso Plus reagent (TaKaRa, Shiga, Japan). Genomic DNA digestion and cDNA synthesis were performed using the ReverTra Ace qPCR RT Kit (TOYOBO, Tokyo, Japan). Quantitative reverse transcription-polymerase chain reaction (qRT-PCR) was performed using the THUNDERBIRD SYBR qPCR Mix (TOYOBO, Tokyo, Japan) with a Thermal Cycler Dic TP800 System (TaKaRa, Shiga, Japan). Serial dilutions of a plasmid containing the open reading frame (ORF) of each gene were used as a standard. The expression levels of the target genes were normalized to an endogenous control *ribosomal protein 49* (*rp49*) in the same sample. Primers amplifying *noppera-bo* (*nobo*), *neverland* (*nvd*), *shroud* (*sro*), *spok*, *phm*, *disembodied* (*dib*), *shadow* (*sad*), and *rp49* have been described previously (McBrayer et al., 2007; Niwa et al., 2010; Enya et al., 2014).

### GFP Reporter Assay

A *spok* promoter-driven GFP reporter assay was performed as described previously (Komura-Kawa et al., 2015). Briefly, *spok>GFP/CyO Act-GFP*; *UAS-deicer2*, *phm22-GAL4/TM6 Ubi-GFP* was established and crossed with *UAS-pabp-IR*. Eggs were laid on grape plates with yeast pastes at 25°C for 2 h. The GFP-negative first instar larvae were picked up and transferred into vials with standard cornmeal food. They were dissected 60 h after egg laying (AEL) and then immunostained.

## Results

### Pabp Plays an Essential Role in the PG During Larval Development

**Figure 1:**
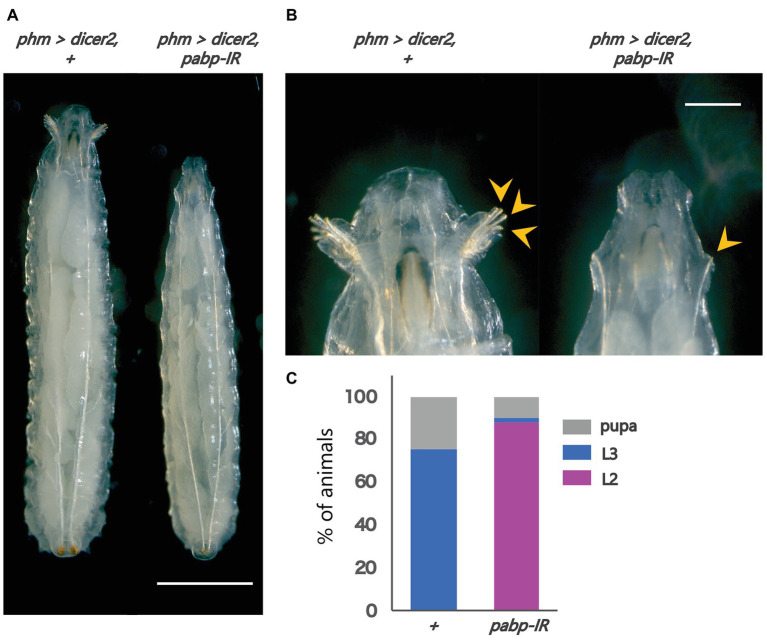
Pabp function is required in the PG for larval development. Phenotypes caused by PG-specific *pabp*-RNAi at 132 h AEL. **(A)** Whole bodies of the control and RNAi larvae. Scale bar: 5 mm **(B)** Magnified intensity of Figure 1A. Scale bar: 2 mm. (Left) *phm>dicer2*, + larvae molted in the third larval instar as judged by the branched morphology of the anterior tracheal pits (yellow arrowheads), typical features of third instar larvae. (Right) *phm>dicer2*, *pabp-IR* larvae raised at the second larval instar and exhibit singular insertions of anterior tracheal pits. **(C)** The developmental progression of *phm>dicer2*, + (*N* = 41) and *phm>dicer2*, and *pabp-IR* (*N* = 49) animals at 132 h AEL. L2: second instar larva, L3: third instar larva.

To examine the importance of Pabp in the PG, we observed the developmental progress of *pabp*-RNAi larvae, for which we used a PG-specific driver (*phm22-GAL4*, hereafter *phm>*) to knock down *pabp* expression by transgenic RNAi. We found that PG-specific *pabp*-RNAi caused a larval-arrest phenotype. Eighty eight percent of *phm>pabp*-RNAi animals were arrested at the second larval instar and even 132 h AEL or later, while only few animals molted into the third larval instar or pupariated at the same time point (Figures 1A–C). The arrested second instar larvae failed to pupariate and died. This result suggests that the Pabp function in the PG is essential for larval development.

**Figure 2:**
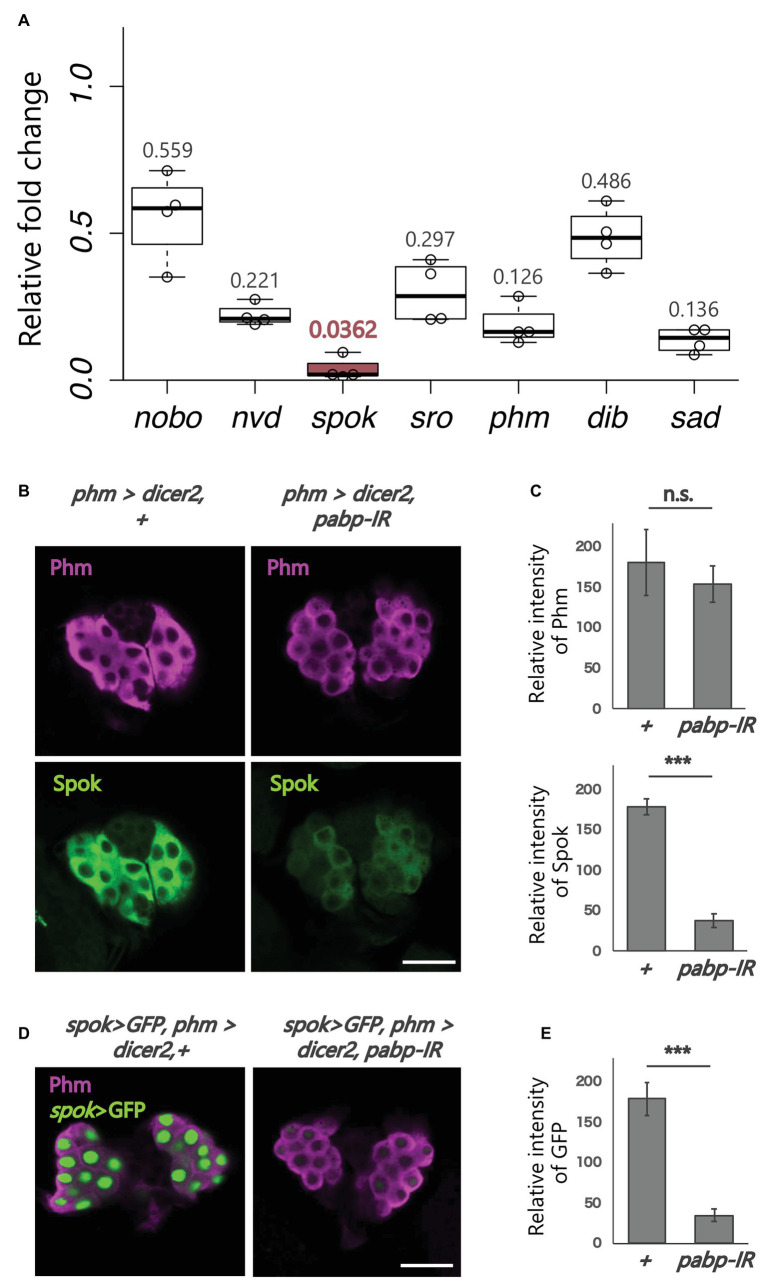
Expression analysis of ecdysteroidogenic genes. All photos are single-plane confocal images. **(A)** Relative expression levels of seven ecdysteroidogenic genes in *phm>dicer2*, *pabp-IR* at 60 h AEL compared to controls (*phm>dicer2*, +) based on the quantitative reverse transcription-polymerase chain reaction (qRT-PCR; *N* = 4). RNA was isolated from the whole bodies of the second instar larvae. **(B)** Immunostaining of the PG cells from *phm>dicer2*, + and *phm>dicer2*, and *pabp-IR* second instar larvae at 60 h AEL with antibodies against Phm (magenta) and Spok (green). **(C)** Quantifications of fluorescence intensities of Phm and Spok in the PG (each *N* = 3). Error bars represent standard deviations. ^***^ and n.s. indicate *p* < 0.001 and non-significance (*p* > 0.05), respectively, by Student’s *t*-test. **(D)** Fluorescence images of the PG cells from *phm>dicer2*, + and *phm>dicer2*, *pabp-IR* larvae with *spok* enhancer/promoter-driven nuclear localized-GFP construct (*spok>GFP*) at 60 h AEL. **(E)** Quantification of GFP fluorescence intensity in the PG nuclei (*N* = 4). The bar plots are drawn in the same manner as **(C)**. PG cells are immunostained with anti-Phm antibody (magenta). Scale bar: 20 μm.

**Figure 3:**
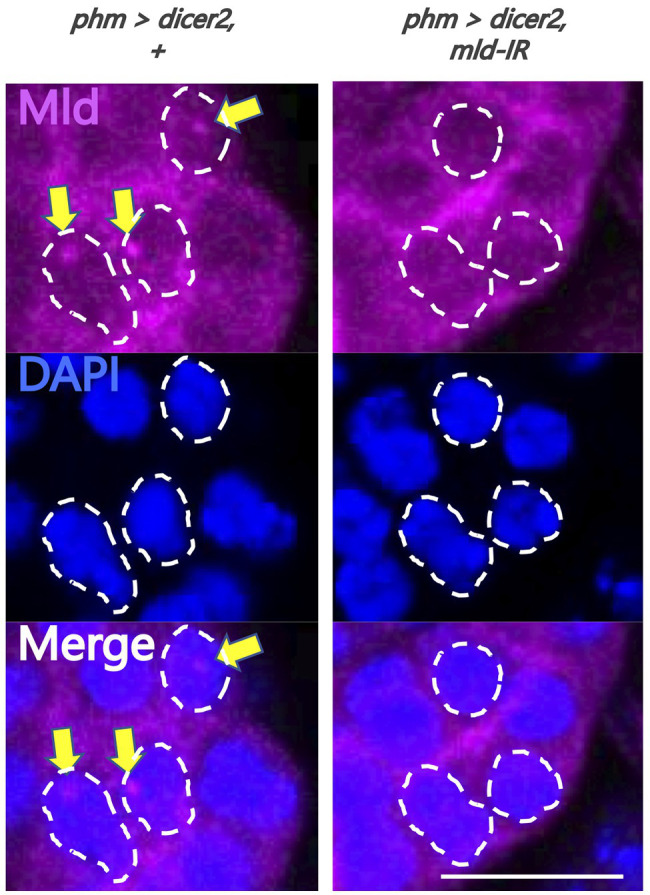
Evaluation of newly-generated anti-molting defective (Mld) antibody. Magnified image of prothoracic gland (PG) cells. All photos are single-plane confocal images. Tissues were stained with anti-Mld antibody (magenta) and 4',6-diamidino-2-phenylindole (DAPI; blue). Representative nuclei are outlined by dashed lines. Mld signals were detected in the nuclei of the PG cells of *phm>dicer2*, + (yellow arrow) at 36 h after egg laying (AEL). In contrast, the signal disappeared in *phm>dicer2* and *pabp-IR*. Scale bar: 10 μm.

### PG-Specific Knockdown of *pabp* Strongly Reduces the Expression of Ecdysteroidogenic Enzyme Genes, Especially *spookier*

Next, we examined whether *pabp* knockdown in the PG changes the expression of ecdysteroidogenic genes. We conducted qRT-PCR to examine the expression levels of seven ecdysteroidogenic genes (Chávez et al., 2000; Warren et al., 2002, 2004; Niwa et al., 2004, 2010; Namiki et al., 2005; Ono et al., 2006; Yoshiyama et al., 2006; Chanut-delalande et al., 2014; Enya et al., 2014) in the second instar larvae of control and *pabp*-RNAi animals. The expression levels of all ecdysteroidogenic genes examined were suppressed in *pabp*-RNAi animals. However, specifically, the suppression levels were different among these seven genes. In particular, the suppression level of *spok*, encoding an ecdysteroidogenic cytochrome P450 enzyme, substantially decreased (Figure 2A). Consistent with this result, the Spok protein level also substantially decreased in the PG of *pabp*-RNAi animals as compared to control animals, while the protein level of another ecdysteroidogenic P450 enzyme Phm was only slightly affected (Figures 2B,C).

To determine whether *spok* expression is transcriptionally and/or translationally disrupted in the *pabp*-RNAi animals, we conducted a GFP reporter assay *in vivo*. The *spok* enhancer region, which is sufficient for *spok* transcription in the PG, has been identified in our previous study (Komura-Kawa et al., 2015). We found that the *spok* enhancer-driven GFP level was almost diminished in PG cells of *pabp*-RNAi animals, suggesting that *pabp* knockdown disrupts *spok* transcription (Figures 2D,E).

**Figure 4:**
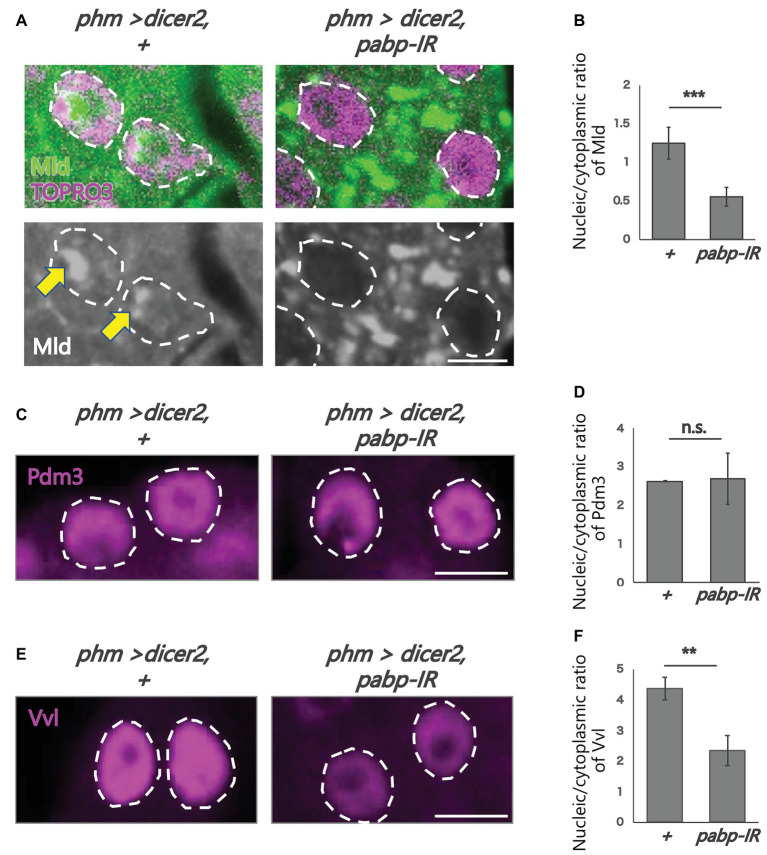
Subcellular localization of transcription factors in the PG cells. All photos are single-plane confocal images. **(A,C,E)** Immunostaining of the PG cells from *phm>dicer2*, + and *phm>dicer2*, *pabp-IR* second instar larvae at 60 h AEL with the antibodies against Mld (green), POU domain motif 3 (Pdm3; magenta), Ventral vein lacking (Vvl; magenta), respectively. Dashed lines indicate the shape of nuclei. **(A)** Nuclei are stained by TOPRO3 (magenta). Bottom panels are single channels of Mld (grayscale). Scale bar: 5 μm. **(B,D,F)** Quantifications of fluorescence intensities of Mld (*N* = 5), Pdm3 (*N* = 3), and Vvl (*N* = 3) in the PG nuclei. Error bars represent standard deviations. ^***^, ^**^ and n.s. indicate *p* < 0.001, *p* < 0.005 and non-significance (*p* > 0.005), respectively, by Student’s *t*-test.

### Reduction of the Expression of *spok* Correlates With Mislocalization of Its Transcriptional Activator Mld

To analyze the molecular mechanism of suppression of *spok* transcription, we focused on the transcription factor Mld. Mld is a zinc-finger type DNA binding protein involved in ecdysone biosynthesis in the PG (Neubueser et al., 2005). Moreover, we have previously reported that Mld is crucial for transcription of *spok*, acting on the upstream region of the *spok* gene locus (Danielsen et al., 2014; Komura-Kawa et al., 2015). In this study, we newly-generated an anti-Mld antibody to visualize Mld protein *in vivo* by immunostaining. Before the immunostaining experiment, we examined the specificity of the newly-generated anti-Mld antibody. We observed that the Mld signal was observed in the nucleus of PG cells in control animals. In contrast, the Mld signal in the PG nucleus disappeared in *mld*-RNAi animals (Figure 3), confirming that the immunostaining signal in the PG nucleus corresponds to Mld protein localization. We then conducted immunostaining with an anti-Mld antibody against *pabp*-RNAi PG cells. Surprisingly, we found that nuclear localization of Mld was disrupted in the *pabp*-RNAi PG cells, although Mld is localized in the nucleus of control PG cells (Figures 4A,B). These results suggest that Pabp regulates the transcription of *spok* by mediating the nuclear localization of Mld.

Next, we examined whether such mislocalization is selective for Mld, but not for other transcription factors in PG cells. To address this issue, we examined the nuclear localization of two other transcription factors such as Vvl and Pdm3, in the *pabp*-RNAi PG cells. Vvl is a POU-domain transcription factor involved in the regulation of the transcription of all known ecdysteroidogenic genes in PG cells (Danielsen et al., 2016). Pdm3 is also a POU-domain transcription factor that is enriched in PG cells (Ou et al., 2016), whereas, its role in PG cells has not yet been elucidated. An immunohistological analysis using anti-Vvl and anti-Pdm3 antibodies revealed that nuclear localization of Vvl and Pdm3 was maintained in the nucleus, even in *pabp*-RNAi PG cells. Nevertheless, the Vvl signal did slightly decrease (Figures 4C–F). Taken together, these results suggest that Pabp regulates the nuclear localization specifically of Mld.

## Discussion

**Figure 5:**
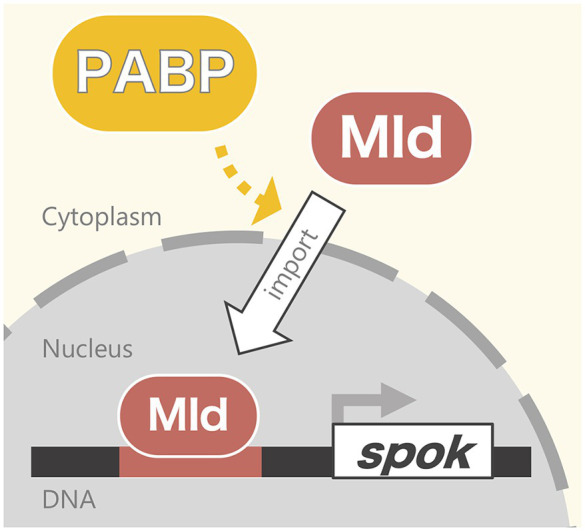
Model of the Pabp function contributing nuclear localization of Mld for *spok* transcription.

In this study, we revealed that Pabp is required for the nuclear localization of the ecdysteroidogenic transcription factor Mld. First, the PG-specific knockdown of *pabp* reduced ecdysteroidogenic gene expression, especially that of *spok*. Second, that reduction of *spok* expression correlated well with the mislocalization of its transcription factor Mld. Third, mislocalization did not occur for all transcription factors but did occur specifically for Mld. In conjunction with our previous data showing that Mld is crucial for inducing *spok* expression through the Mld-response element in *spok* promoter region (Uryu et al., 2018), we propose that Pabp positively regulates *spok* expression *via* mediating nuclear localization of Mld in PG cells (Figure 5).

The predicted ORF of *mld* encodes a protein that belongs to the family of the zinc-finger associated domain (ZAD) containing C_2_H_2_ zinc-finger proteins (ZFPs; Neubueser et al., 2005). A previous study has shown that modification or depletion of ZAD disrupts nuclear localization of ZAD-ZFPs (Zolotarev et al., 2016). In conjunction with our observation, this fact raises the possibility that ZAD may be involved in the Pabp-dependent nuclear localization of ZAD-ZFPs, whereas, this hypothesis has yet been experimentally examined. It would also be intriguing to examine whether the subcellular localization of Ouija board (Ouib) and Séance (Séan), other ZAD-ZFP transcription factors of *spok* and *nvd*, respectively (Komura-Kawa et al., 2015; Uryu et al., 2018), are also affected by *pabp*-RNAi. Currently, we failed to generate anti-Ouib and anti-Séan specific antibodies.

This is the first report showing a novel function of Pabp in controlling the nuclear localization of a transcription factor for ecdysone biosynthesis. In the last decade, several transcription factors for ecdysone biosynthesis have been identified (Niwa and Niwa, 2016), while the regulation of subcellular localization of these transcription factors has not been rigorously studied; except DHR4 (Ou et al., 2011). Pabp and other poly(A)-related proteins might be critical research targets for understanding the nuclear and cytoplasmic translocation of ecdysteroidogenic transcription factors in the future.

## Data Availability Statement

The raw data supporting the conclusions of this article will be made available by the authors, without undue reservation, to any qualified researcher.

## Author Contributions

Study conception: TK, WS, and RN. Investigation (performing of the experiments): TK, NT, and RN. Investigation (data analysis): TK, WS, NT, AN, and RN. Writing/manuscript preparation (writing the initial draft): TK and RN. Writing/manuscript preparation (critical review, commentary, or revision): TK, WS, NT, AN, and RN. Funding acquisition: AN and RN. All authors contributed to the article and approved the submitted version.

## Conflict of Interest

The authors declare that the research was conducted in the absence of any commercial or financial relationships that could be construed as a potential conflict of interest.
